# Developing and testing an instrument for identifying performance incentives in the Greek health care sector

**DOI:** 10.1186/1472-6963-6-118

**Published:** 2006-09-13

**Authors:** Victoria Paleologou, Nick Kontodimopoulos, Aggeliki Stamouli, Vassilis Aletras, Dimitris Niakas

**Affiliations:** 1 Faculty of Social Sciences, Hellenic Open University, Riga Fereou 169 & Tsamadou 26222 Patras, Greece

## Abstract

**Background:**

In the era of cost containment, managers are constantly pursuing increased organizational performance and productivity by aiming at the obvious target, i.e. the workforce. The health care sector, in which production processes are more complicated compared to other industries, is not an exception. In light of recent legislation in Greece in which efficiency improvement and achievement of specific performance targets are identified as undisputable health system goals, the purpose of this study was to develop a reliable and valid instrument for investigating the attitudes of Greek physicians, nurses and administrative personnel towards job-related aspects, and the extent to which these motivate them to improve performance and increase productivity.

**Methods:**

A methodological exploratory design was employed in three phases: a) content development and assessment, which resulted in a 28-item instrument, b) pilot testing (N = 74) and c) field testing (N = 353). Internal consistency reliability was tested via Cronbach's alpha coefficient and factor analysis was used to identify the underlying constructs. Tests of scaling assumptions, according to the Multitrait-Multimethod Matrix, were used to confirm the hypothesized component structure.

**Results:**

Four components, referring to intrinsic individual needs and external job-related aspects, were revealed and explain 59.61% of the variability. They were subsequently labeled: *job attributes*, *remuneration*, *co-workers *and *achievement*. Nine items not meeting item-scale criteria were removed, resulting in a 19-item instrument. Scale reliability ranged from 0.782 to 0.901 and internal item consistency and discriminant validity criteria were satisfied.

**Conclusion:**

Overall, the instrument appears to be a promising tool for hospital administrations in their attempt to identify job-related factors, which motivate their employees. The psychometric properties were good and warrant administration to a larger sample of employees in the Greek healthcare system.

## Background

Conventional human resources theories, developed more than 50 years ago by Maslow and Herzberg, suggest that satisfied employees tend to be more productive, creative and committed to their employers. Organizational features such as policies and procedures, leadership style, operations, and general contextual factors of the setting, all have a profound effect on how staff perceives the quality of work life, and in creating a motivating and satisfying work environment [1]. These features are prerequisites for retaining an adequate and qualified workforce and an overall well-operating organization. In the domain of organizational behavior, job satisfaction research is not a new issue and various studies have attempted to measure its effect on organizational performance [2,3].

In a world of resource shortages, the healthcare sector faces this limitation more than other industries due to the decrease of skilled labour, the high cost and increased complexity of technology, increased demand from the aging population, increased regulations, regulatory compliance and demand of services for continuous quality improvement, the consumer orientation of the industry, and various ongoing reorganizations [4]. These factors imply that the concepts of productivity, job satisfaction, and motivation have become very important in retaining a well-performing staff.

In Greece, national legislation, enforced in 2001, clearly identifies technical and economic efficiency as major improvement targets for the health system. Hospital administrations are accountable not only for the effectiveness of provided services, but for their efficiency as well. Keeping in mind that increased technical and economic efficiency usually implies reduction of resources, it becomes obvious that staff performance, as perhaps the most important factor in a labor-intense environment, should be a major improvement target. Hospital administrations, particularly in the public sector, are limited in their ability to provide additional financial incentives, which could motivate employees to perform better. Therefore it is important to understand what else satisfies health professionals in their workplace, and motivates them to improve performance.

Within this context, the purpose of this study was twofold. Initially, to develop an instrument suitable for measuring employee motivation in the health care sector, which could eventually be adopted and used by hospital administrators in an attempt to identify particular work-related factors that motivate their staff to perform better. Following this, to measure its basic psychometric properties, namely reliability and validity via administration to a representative sample of workers of the Greek health system.

### Theories of motivation

Motivation is an internal driving force that is not easily influenced by external factors. However, managers can satisfy employees so they become motivated and of all the functions a manager performs, motivating employees is arguably the most complex, since motivation is influenced by both financial and non-financial incentives [5] due, in part, to the fact that what motivates employees changes constantly [6]. The terms *job satisfaction *and *motivation *are often used interchangeably, however there is a borderline. Job satisfaction is a person's emotional response to his or her job condition, whereas motivation is the driving force to pursue and satisfy needs. The need for motivation stems from the need for survival and motivated employees help organizations survive [7].

Motivational theories can be divided into two categories, *content *and *process *theories. The former assume that all individuals possess the same set of needs and therefore prescribe the characteristics that should be present in jobs, while the latter stress the difference in people's needs and focus on the cognitive processes that create these differences. We will briefly describe two well-known content theories, *Maslow's need-hierarchy theory *and *Herzberg's two-factor theory*, which influenced the choice of items in the measuring instrument used in this study.

According to Maslow, employees have five levels of needs (physiological, safety, social, self-esteem, and self-actualising) and he argued that lower level needs have to be satisfied before the next higher level need could motivate [8]. This theory was not intended as an explanation of motivation in the workplace, however many managerial theorists have adopted it. Herzberg's work categorized motivation into two factors, *motivators *and *hygienes*. Motivators such as responsibility, achievement, recognition, promotion and various intrinsic aspects cause states of motivation. Hygiene factors such as supervision, salary, work environment and relationships do not increase job satisfaction, however their absence causes job dissatisfaction [9].

### Job satisfaction

The expected behavioural correlate of job satisfaction is higher work performance, however research has not been able to establish a strong link between the two, and meta-analytic correlations do not support the assertion that the latter is heavily dependent on the former [10]. The knowledge, skills and abilities of the workforce, the situation as well as the technology or equipment available all have a greater influence on performance than job satisfaction. However job satisfaction and motivation work together to increase job performance and healthcare organizations can do many things to increase job satisfaction, primarily by focusing on the motivating interests of existing and future staff [11]. It has been shown that low job satisfaction is a major cause of turnover among health care providers [12-14], that it affects the quality of service and organizational commitment [15,16] and may be associated with staff shortages [17,18] or psychosocial stress [19].

In the health care sector, few organizations have made job satisfaction a top priority, perhaps because they have failed to understand the significant opportunity that lies in front of them. Satisfied employees tend to be more creative and committed to their organizations, and recent studies have shown a direct correlation between staff satisfaction and patient satisfaction [20]. Hospital administrators who can create work environments that attract, motivate and retain hard-working individuals will be better positioned to succeed in a competitive health care environment demanding quality and cost-efficiency. What's more, they may even discover that by creating a positive workplace for their employees, they've increased their own job satisfaction as well [21].

## Methodology

### Instrument development

Following a systematic Medline review of the international literature pertinent to the subject in question, studies reporting on multidimensional job satisfaction instruments were collected on the basis of theoretical models that integrate the findings of empirical research related to job satisfaction and motivation. The search terms used were combinations of "job satisfaction", "motivation", "employee" and "instruments" in the title or the abstract and they were elaborated by combining synonyms and similar words. To restrict the number of studies, general inclusion criteria were set. The timeframe of the search was publication between 1995 and 2005, peer-reviewed and written in English. Next the instruments were assessed on the basis of their minimum psychometric characteristics, specifically reliability and construct validity, according to the results reported in each study. Providing these properties had been previously demonstrated, they were further examined for responsiveness and content validity [22]. This strategy eventually identified 8 studies about instruments for heterogeneous populations [23-30] and 10 more for specific job populations in the health care sector [31-40].

The questionnaires and scales used in these studies were the basis for constructing the current instrument. The forty-eight most relevant, to the purposes of this study, questions were translated and adapted to the Greek cultural context. They were arranged in a manner that would secure the desired information without confusing the interviewees. Closed-ended questions were used because they are easier to answer, require less time, help to focus on the subject under question, increase willingness to participate, enhance objectivity and are easier to codify and perform quantitative analysis. Careful attention was paid to question phrasing in order to create a clear and complete, yet concise, instrument that would achieve high response rates and be free of errors and biases during completion and processing.

To increase confidence that the instrument covers relevant theoretical framework, or at least a significant portion of it, item selection was guided by Maslow and Herzberg's theories. To further enhance content validity, three experts in human resource management and psychometry reviewed the first draft of the instrument. They were individually asked to judge the questions for appropriateness, clarity and completeness and the instrument in its entirety for appearance, question sequence and completion time. The procedure was then repeated with three more experts. The result of these two expert reviews was the identification of questions overlapping in construct, others that were vague, ambiguous and redundant and some, which appeared irrelevant to the objectives of the study, all of which were removed, resulting in a twenty-eight item instrument.

Next, the shortened version was pilot-tested in an Athens-based general hospital, using a random sample of 74 hospital employees, equally comprised of physicians, nurses and administrative staff (this category includes not only office workers, but all hospital employees other than physicians and nurses). At this stage the instrument was administered via a semi-structured interview to ensure that additional qualitative data, which could help enhance the instrument, would not be lost. Furthermore this would increase validity control through constant observation of the interviewees. Frequently, the combination of quantitative and qualitative data is used when the phenomena in question are complex and require in-depth study of many aspects [41].

The pilot-testing showed that the instrument was simple and straightforward to complete, not time-consuming (approximately 15 minutes) and generally accepted by the interviewees. It comprised of two parts. The first contained a standard set of sociodemographic and job related questions addressing age, sex, education, position, years of experience and department. The second part contained twenty-eight questions addressing intrinsic and extrinsic motivators. Intrinsic are those characteristics inherent in the job, including autonomy, professional growth, group cohesion and professional characteristics. Extrinsic motivators are those external to the job such as salary, rewards, working conditions, flexibility, stability and workload. All questions were neutrally phrased as "*In your case, how important is... .... for increasing your will to perform better at work?*". Responses were provided on a five-point unipolar adjective scale, in which 1 corresponded to *"not at all"*, 2 to *"a little bit"*, 3 to *"moderately"*, 4 to *"very" *and 5 to *"extremely"*.

### Sample and data collection

To investigate the suitability of the instrument in the Greek healthcare environment, a large and fairly representative sample of professionals was recruited. The testing fields were five general hospitals of the National Health System, located in the greater Athens area. The survey was reviewed and approved by the hospital Review Boards and permission to administer to employees was granted by the administrations. The study was conducted between February and April 2006 and 450 questionnaires were self-administered to a random sample of 100 physicians, 200 nurses and 150 administrative personnel, in order to represent the distribution of these groups within the Greek health system. The response rates were physicians: 83%, nurses: 81% and administrative personnel: 72% and overall 77.8%, i.e. 353 questionnaires were eventually collected. Instructions were provided via an accompanying letter describing the purpose of the study and stating that participation was voluntary and anonymous.

### Analysis

The sample was analyzed as a whole, i.e. all 353 questionnaires. Item descriptive statistics and the response distribution for each item were calculated, in order to examine central tendency, variability and symmetry. Reliability and validity, as main psychometric properties, were investigated. Specifically, internal consistency reliability, i.e. how well items reflecting the same construct yield similar results, was tested via Cronbach's alpha coefficient which ranges in value from 0 to 1 and is the most frequently used estimate of internal consistency. The higher the score, the more reliable the generated scale is, meaning that its items demonstrate a high degree of inner-correlation. It has been indicated that 0.70 is an acceptable reliability coefficient [42] but lower thresholds are sometimes used in the literature.

Validity, i.e. the degree to which the instrument measures what it claims to be measuring, was tested via content and construct validity. Content validity was demonstrated by assessing if the instrument was a representative sample of the content it was originally designed to measure and this was addressed in the development stage. Specifically, the literature review aimed to identify the broadest possible group of similar instruments used in related studies and to exploit upon the experience of other researchers and experts. This helped to increase confidence that important aspects of job satisfaction generating motivation had not been omitted. The content was reviewed two times by national experts to ensure its adaptability to the local cultural context.

To support construct validity, factor analysis was used to determine the underlying constructs, which explain significant portions of the variance in the instrument items. The factor loadings, i.e. the correlation coefficients between the items and factors, were examined in order to explain the meaning of each construct. Tests of scaling assumptions, according to the Multitrait-Multimethod Matrix [43], were used to confirm the hypothesized structure. These include item internal consistency which is substantial and satisfactory when correlation between an item and its hypothesized scale (corrected for overlap) is at least 0.40 and item discriminant validity which is successful when the correlation between an item and its own scale is significantly higher, by two standard errors or more, than with other scales [44]. All analyses were performed using SPSS software, version 12.0 (SPSS Inc., Chicago IL).

## Results

The group of 353 hospital employees responding to the survey was comprised of 83 physicians, 162 nurses and 108 administrative staff. The majority was female (69.5%) and the mean age for the whole sample was 39.3 years. The mean time spent in the particular hospital was 11.2 years and in the current position 8.0 years. Also, 24.4% of respondents were responsible for managing other people. Information pertinent to the responses is provided in Table 1, which displays the item descriptive statistics. Valid response rates are very high for all items, providing evidence that items and response choices are clear and unambiguous. Furthermore, all response choices were used in every case.

The suitability of the data for factor analysis was tested via the Kaiser-Meyer-Olkin (KMO) measure of sampling adequacy, which tests the partial correlations among the items, and its value should be greater than 0.5 for a satisfactory factor analysis to proceed [45]. The result in this study for the KMO measure was 0.929. Next, Bartlett's test of sphericity demonstrated that the correlation matrix was not an identity matrix, implying the appropriateness of the factor model (P < 0.0005). Principal Component Analysis (PCA) was chosen as the extraction method for obtaining the initial factor solution.

A decision to be made in factor analysis is the number of factors and a typical approach is the Kaiser-Guttman rule which states that an eigenvalue (i.e. the variance accounted for by each factor) of greater than one is the only criterion required because it wouldn't make sense to add a factor that explains less variance than is contained in one variable. However, this approach usually produces many factors along with the inherent difficulty of properly interpreting each one. Another eigenvalue-based approach is to examine Cattell's "scree" plot, i.e. a two-dimensional graph with factors on the x-axis and "eigenvalues" on the y-axis, by looking for a spot in the plot where it abruptly levels out. By employing both methods, four factors were identified in this study.

Varimax orthogonal rotation, which assumes that the factors are uncorrelated with one another, simplified their interpretation by minimizing the items with high loadings on each factor. This maximizes high loadings and minimizes low loadings so that the simplest possible structure is achieved. Factor analysis demonstrated four factors cumulatively accounting for 59.61% of variation in all the items. Specifically, the first factor accounts for 18.67% of the variability, the second for 15.15%, the third for 14.60%, and the fourth for 11.19%.

Table 2 represents the rotated component matrix of item loadings on each of the four factors, and the squared factor loading is the percent of variance in that item explained by the factor. A cut-off point of 0.50 for factor loadings was adopted, i.e. only those items correlating with their hypothesized factor above this threshold were retained for further analysis [46]. This criterion resulted in dropping four items from further analysis, namely *"job security", challenging and interesting work", "clear responsibilities" *and *"recognition of good performance"*.

Cross-loading items, i.e. those items loading at 0.32 or higher (equating to approximately 10% overlapping variance with other items in that factor) were also left out [47]. Cross-loading is an indication that the item may be poorly written and it could affect the factor structure, and can also raise questions about convergent and discriminant validity. However, to avoid removing more items than necessary, a second criterion was introduced, by which items with a factor loading difference of at least 0.2, independent of cross-loading, were retained [48]. These two criteria combined resulted in the removal of five more items, specifically, *" growth and development", "achievement-related promotion", "adequate means and equipment"*, *"physical safety" *and *"freely expressing opinions"*.

The 19 items retained in the analysis were used to create the multitrait/multi-item correlation matrix shown in Table 3, which helps to examine the relationship of each item with its hypothesized scale, as well as its correlations with other scales. Each row in the matrix shows correlations between the responses for one item and all hypothesized item groupings and each column contains correlations between one scale and all items in the matrix, including those hypothesized to be part of that scale, and those hypothesized to be part of other scales. The correlation between an item and its scale is corrected for overlap, i.e. that item is removed from the scale. Item internal consistency is satisfactory and the criterion of 0.40 or more correlation of an item and its hypothesized scale is satisfied for all items. Cronbach's alpha coefficient is high in all scales, ranging from 0.782 to 0.901, well above the 0.70 recommended level.

The multitrait/multi-item correlation matrix also allows examination of the assumption that items are stronger measures of their hypothesized constructs than of other constructs (test of item discriminant validity). Table 4 summarizes the results of the discriminant validity tests performed in this study. Results were categorized in two groups according to the degree of discrimination. More specifically, "level 1" success is defined as item-scale correlation that is higher for the hypothesized scale than for competing scales. On the other hand, "level 2" is designated as item-scale correlation higher for the hypothesized scale than competing scales by two standard errors or more, where the standard error of the correlation coefficient is approximately equal to 1 divided by the square root of the sample size. Specifically, 57 cases were examined, i.e. the correlation of each of the 19 items with its hypothesized scale compared to the correlation with the three competing scales. Discriminant validity tests, for all four factors, predominantly demonstrated level-2 (significant) success, implying that each item, within a scale, does not measure unrelated constructs. The final and perhaps most difficult step is interpreting the factors emerging from this analysis and this is attempted in the next section.

## Discussion

This study reported on the development and psychometric testing of an instrument for measuring what motivates employees in the healthcare sector. This constitutes the first stage of a broader study underway, aiming to identify factors that motivate workers and lead to increased job productivity in the Greek health care system. Within a long-term perspective, this information could help hospital management increase overall performance, both individual and organizational. The theoretical framework of the study rests on well-known motivation theories that have supported similar efforts over the years.

The draft version of the instrument was based on the results of an extensive international literature review and contained 48 questions, which were translated and culturally adapted for the Greek population. This initial format was subjected to two review stages in which experts assessed the instrument for its overall appropriateness, clarity and completeness and other issues such as appearance, question sequence and completion time. This exercise was an important step for supporting content validity and resulted in a shortened version containing 28 questions to be pilot-tested by a small group (N = 74) of employees in a large Athens-based hospital. The results of this small-scale assessment did not reveal significant problems, and the next important step was to assess the instrument's reliability and validity, via field-testing with a large and representative sample (N = 353).

Principal component analysis was performed and, after varimax rotation, four factors were extracted accounting for 59.61% of the variance of the items. Item-scale criteria were not satisfied in the case of nine items, which were eventually excluded from the instrument. Construct validity was supported by the overall success of the convergent and discriminant validity tests of item-scale correlations, according to the Multitrait-Multimethod Matrix approach. The four factors represent distinct constructs of motivation, addressed by the instrument according to the results of the psychometric analysis. Reliability was addressed via internal consistency reliability using Cronbach's alpha coefficient, which was well above the recommended minimum value for each individual construct.

The first factor, which was labeled *job attributes*, contains 7 items and addresses motivators linked to particular job characteristics such as participation in decision-making, creativity and skill exploitation. These are intrinsic motivators, i.e. they address self-needs generated from individual internal values that must be satisfied before experiencing true job satisfaction. The second factor consists of four items and was labeled *remuneration*. In this case, extrinsic work-related motivators are addressed, such as salary and benefits, pension, insurance, etc. The third factor consists of five items and was labeled *co-workers*. It refers to professional relationships with supervisors and colleagues as a source of satisfaction and potential motivation. Finally, the fourth factor consists of three items and was labeled *achievement*. This also refers to intrinsic motivators expressed through factors such as pride, appreciation, respect and social acceptance.

To further interpret the factors, we attempt to link them to the content motivation theories discussed earlier. They appear to reflect on different levels of Maslow's *"hierarchy of needs"*. Specifically, the items under the factor *job attributes *are associated with motivators such as accomplishment, creativity, and growth, i.e. *self-esteem *according to Maslow's terminology. These needs are caused by the work itself and they do not depend on how others value or acknowledge an employee's effort. When these needs exist, they are among the strongest of internal motivators. By providing employees with projects that challenge their strengths, utilize their talents, and help them develop new skill sets, mangers can help them be successful. However, caution is suggested when providing creative challenges to those who do not have these needs.

The factor *remuneration *clearly reflects safety needs such as security and stability, which are met by providing a safe working place, good benefits including retirement, and insurance, all of which are motivators that advance employee welfare and ensure that future needs are met. Accordingly, the third factor *co-workers*, includes items related to social needs such as belongingness, relationships and acceptance in formal and informal work groups. This is equivalent to the third level on Maslow's pyramid, and a comfortable work environment, together with open communication can provide these necessities. As for *achievement*, there is an apparent association with Maslow's highest level of needs, i.e. self-actualization. After meeting the previous levels of need, a person will pursue self-actualization, take risks, learn new things, reach one's full potential and generally grow in the work environment.

According to Herzberg's two-factor theory, factors *remuneration *and *co-workers *are hygiene factors. While these do not motivate, they can satisfy employees if handled properly. On the other hand, factors *job attributes *and *achievement *are motivation factors because they create satisfaction by fulfilling an individual's higher needs. Once hygiene factors are met, the motivation factors will, according to Herzberg, promote job satisfaction and encourage production.

A limitation of this study is that the instrument was tested for its overall psychometric properties using the combined sample of physicians (N = 83), nurses (N = 182) and administrative personnel (N = 108). Given the huge differences in job conditions, work pressures and salaries, across lower status administrative personnel to high status physicians, it would be unrealistic to claim that this instrument is useable as a general survey instrument for Greek hospitals. To support this assertion, a larger sample is required for testing between and within groups.

The problems and solutions to motivation issues can be complex, and the timeless theories of Maslow, Herzberg and others, despite not ever having received any empirical support from research, can offer ideas and solutions to motivation problems. However, motivation management and the individual motivation profile are also useful tools for self-analysis and discovering how to motivate certain individuals or groups. Managers that utilize these tools and ideas can be successful motivators. Improving motivation usually starts with setting high organizational expectations and by knowing an individual's profile, a manager can then tailor a motivation method for that person. Maslow and Herzberg both argued that their respective theories applied to everyone, whereas modern motivation researchers recognize a wide range of individual differences, rather than one universal approach [49,50].

## Conclusion

This study resulted in the development of an instrument for measuring employee motivation, to be eventually used in the healthcare sector in Greece. After psychometric testing it demonstrated acceptable levels of internal consistency reliability and content and construct validity, according to standards quoted in the literature. This evidence warrants a large-scale administration of the instrument to hospital employees. This will help to confirm the psychometric properties reported here, both between and within the various groups. More importantly it will be the basis for investigating what motivates groups of healthcare professionals, in an attempt to increase productivity in a very sensitive and critical sector of public services.

## Competing interests

The author(s) declare that they have no competing interests.

## Authors' contributions

VP was responsible for designing the study, conducting the literature review, developing the questionnaire and acquiring the data. NK was responsible for analyzing and interpreting the data and drafting the manuscript. AS conducted the statistical analysis and helped to finalize the questionnaire. VA assisted in interpreting the results and revising the manuscript for intellectual content. DN was responsible for conception of the study. All authors have read and approved the final manuscript.

## Pre-publication history

The pre-publication history for this paper can be accessed here:



## Figures and Tables

**Table 1:** Item descriptive statistics

**Item**	**Description**	**Valid (%)**	**Mean (SD)**	**Median**	**Distribution of valid responses (%)**				
					
					**1**	**2**	**3**	**4**	**5**
1	Salary and fringe benefits	99.7	3.53 (1.49)	4.0	13.9	15.3	15.9	13.1	41.8
2	Environmental conditions	100.0	3.93 (1.24)	4.0	7.1	7.1	16.4	24.6	44.8
3	Job security	100.0	4.24 (1.13)	5.0	4.8	4.5	11.9	19.0	59.8
4	Retirement and insurance	100.0	3.90 (1.24)	4.0	5.7	9.9	18.4	21.2	44.8
5	Absence policies	99.7	3.74 (1.31)	4.0	8.8	9.9	19.0	23.0	39.3
6	Freely expressing opinions	100.0	3.85 (1.22)	4.0	7.4	7.4	16.4	30.9	37.9
7	Job meaningfulness	100.0	4.29 (1.06)	5.0	3.4	5.1	9.6	23.3	58.6
8	Respected as a person	100.0	4.33 (1.03)	5.0	4.0	2.3	11.6	21.0	61.1
9	Growth and development	100.0	3.78 (1.30)	4.0	8.5	9.6	17.1	24.9	39.9
10	Strong interpersonal relations	100.0	4.24 (0.97)	5.0	2.0	4.5	12.5	29.7	51.3
11	Achievement-related promotion	100.0	3.54 (1.38)	4.0	13.3	9.6	19.8	24.4	32.9
12	Adequate means and equipment	100.0	3.83 (1.20)	4.0	5.1	11.0	17.8	28.1	38.0
13	Recognition of good performance	100.0	4.00 (1.23)	4.0	6.8	6.5	14.7	24.1	47.9
14	Team-work	100.0	3.50 (1.15)	4.0	7.1	9.6	31.4	29.5	22.4
15	Job-related pride and respect	100.0	3.80 (1.19)	4.0	5.9	9.1	19.5	29.5	36.0
16	Physical safety	100.0	3.80 (1.24)	4.0	7.1	8.5	20.1	26.1	38.2
17	Role appreciated	99.7	3.75 (1.19)	4.0	6.8	7.1	23.6	29.5	33.0
18	Challenging and interesting work	99.7	4.01 (1.10)	4.0	4.0	6.8	15.9	30.7	42.6
19	Supervisor support	99.4	3.92 (1.25)	4.0	8.0	7.4	12.0	30.2	42.4
20	Exercising authority	100.0	2.75 (1.30)	3.0	23.5	18.7	27.8	19.3	10.7
21	Significant goals	100.0	3.21 (1.34)	3.0	15.0	16.2	21.8	26.9	20.1
22	Treated fairly	99.4	3.97 (1.21)	4.0	6.3	8.0	13.1	28.2	44.4
23	Creativity opportunities	100.0	3.54 (1.27)	4.0	9.3	11.9	21.8	28.9	28.1
24	Clear duties and objectives	100.0	3.67 (1.24)	4.0	6.8	13.3	17.3	30.9	31.7
25	Control over job decisions	99.7	3.49 91.39)	4.0	13.4	11.6	20.2	22.7	32.1
26	Skill exploitation	100.0	3.80 (1.15)	4.0	5.1	9.1	20.4	31.4	34.0
27	Clear responsibilities	100.0	4.16 (1.02)	4.0	2.8	4.5	14.2	30.6	47.9
28	General decision-making	100.0	3.33 (1.40)	3.0	15.9	12.2	22.4	22.4	27.1

**Table 2:** Rotated component matrix (factor loadings)

**Item**	**Description**	**Factor**			
		
		**1**	**2**	**3**	**4**
9	Growth and development	**0,576**	0,429	-0,039	0,463
11	Achievement-related promotion	**0,581**	0,442	-0,058	0,421
20	Exercising authority	**0,589**	0,227	0,313	-0,131
21	Significant goals	**0,754**	0,248	0,247	0,086
23	Creativity opportunities	**0,708**	0,096	0,236	0,286
24	Clear duties and objectives	**0,621**	0,254	0,380	0,127
25	Control over job decisions	**0,743**	0,186	0,178	0,241
26	Skill exploitation	**0,651**	0,147	0,326	0,292
28	General decision-making	**0,667**	0,203	0,257	0,198
1	Salary and fringe benefits	0,276	**0,767**	0,010	0,175
2	Environmental conditions	0,209	**0,738**	0,132	0,127
3	Job security	-0,058	**0,437**	0,404	0,150
4	Retirement and insurance	0,169	**0,697**	0,202	0,133
5	Absence policies	0,075	**0,706**	0,281	0,169
12	Adequate means and equipment	0,363	**0,554**	0,165	0,245
16	Physical safety	0,374	**0,515**	0,477	0,050
14	Team-work	0,229	0,113	**0,600**	0,135
15	Job-related pride and respect	0,248	0,152	**0,708**	0,156
17	Role appreciated	0,347	0,233	**0,634**	0,131
18	Challenging and interesting work	0,454	-0,111	**0,467**	0,280
19	Supervisor support	0,184	0,181	**0,706**	0,152
22	Treated fairly	0,125	0,234	**0,664**	0,198
27	Clear responsibilities	0,311	0,016	**0,466**	0,334
6	Freely expressing opinions	0,332	0,426	0,139	**0,537**
7	Job meaningfulness	0,226	0,057	0,315	**0,626**
8	Respected as a person	0,154	0,274	0,219	**0,771**
10	Strong interpersonal relations	0,115	0,299	0,322	**0,664**
13	Recognition of good performance	0,348	0,398	0,235	**0,480**

**Table 3:** Item-factor correlations corrected for overlap

**Item**	**Description**	**FACTOR**			
		**1**	**2**	**3**	**4**
	**1^st ^FACTOR: 7 Items (alpha = 0.901)**				
20	Exercising authority	**0.558**	0.376	0.456	0.293
21	Significant goals	**0.734**	0.443	0.518	0.396
23	Creativity opportunities	**0.736**	0.378	0.484	0.477
24	Clear duties and objectives	**0.722**	0.465	0.541	0.443
25	Control over job decisions	**0.762**	0.429	0.459	0.458
26	Skill exploitation	**0.731**	0.423	0.549	0.475
28	General decision-making	**0.724**	0.438	0.495	0.441
				
	**2^nd ^FACTOR: 4 Items (alpha = 0.822)**				
1	Salary and fringe benefits	0.442	**0.708**	0.343	0.409
2	Environmental conditions	0.420	**0.654**	0.369	0.406
4	Retirement and insurance	0.416	**0.604**	0.360	0.415
5	Absence policies	0.410	**0.626**	0.442	0.426
				
	**3^rd ^FACTOR: 5 Items (alpha = 0.826)**				
14	Team-work	0.406	0.287	**0.535**	0.362
15	Job-related pride and respect	0.491	0.335	**0.684**	0.483
17	Role appreciated	0.554	0.406	**0.630**	0.446
19	Supervisor support	0.480	0.344	**0.647**	0.402
22	Treated fairly	0.454	0.402	**0.613**	0.420
				
	**4^th ^FACTOR: 3 Items (alpha = 0.782)**				
7	Job meaningfulness	0.436	0.314	0.427	**0.550**
8	Respected as a person	0.453	0.476	0.450	**0.700**
10	Strong interpersonal relations	0.440	0.491	0.504	**0.616**

Each item-factor correlation corrected for overlap (relevant item removed). Bold text correlations are hypothesized to be highest in same row.

**Table 4:** Summary results of convergent and discriminant validity tests

				**Item-Discriminant Validity**					
				
		**Item-Internal Consistency**		**Range of Correlations^4^**				**Test success^5^**	
				
**Factors**	**N^1^**			**Factors**					
		**Range of Correlations^2^**	**# Success/Total^3^**	**1**	**2**	**3**	**4**	**Level 1**	**Level 2**
**1**	7	0.558–0.762	7/7	---	0.376–0.465	0.456–0.549	0.293–.0477	1/21	20/21
**2**	4	0.604–0.708	4/4	0.410–0.442	---	0.343–0.442	0.406–0.426	0/12	12/12
**3**	5	0.535–0.684	5/5	0.406–0.554	0.287–0.406	---	0.362–0.483	1/15	14/15
**4**	3	0.550–0.700	3/3	0.436–0.453	0.314–0.491	0.427–0.504	---	0/9	9/9

^1 ^Number of items, ^2 ^Range of correlations between items and hypothesized factor corrected for overlap, ^3 ^Number of correlations exceeding the 0.40 standard/total number of correlations, ^4 ^Range of correlations between items and other factors, ^5 ^Successful discriminant validity tests. Level 1: Item-factor correlation is higher for hypothesized scale than competing scales. Level 2: Item-scale correlation is significantly higher for hypothesized scale than competing scales. The default significance level for comparing two correlations is two standard errors. The standard error of a correlation coefficient is approximately equal to 1 divided by the square root of the sample size.
